# *Col6a1* knock-in mice provide a promising pre-clinical model for collagen VI-related dystrophies

**DOI:** 10.1242/dmm.052460

**Published:** 2026-01-22

**Authors:** Arístides López-Márquez, Carmen Badosa, Lluis Enjuanes-Ruiz, Patricia Hernández-Carabias, Manuel Sánchez-Martín, Bruno Cadot, Zoheir Guesmia, Ioannis Georvasilis, Sol Balsells, Marcos Blanco-Ramos, Emma Puighermanal, Albert Quintana, Mònica Roldán, Valérie Allamand, Cecilia Jiménez-Mallebrera

**Affiliations:** ^1^ Neuromuscular Unit, Neuropaediatrics Department, Institut de Recerca Sant Joan de Déu, Hospital Sant Joan de Déu, Esplugues de Llobregat, 08950 Barcelona, Spain; ^2^ Center for Biomedical Research on Rare Diseases (CIBERER), Instituto de Salud Carlos III, 28029 Madrid, Spain; ^3^ Department of Genetics, Microbiology and Statistics, University of Barcelona, 08028 Barcelona, Spain; ^4^ Instituto de Investigación Biomédica de Salamanca (IBSAL), 37007 Salamanca, Spain; ^5^ Servicio de Transgénesis, Department of Medicine, Universidad de Salamanca, 37007 Salamanca, Spain; ^6^ Sorbonne Université-Inserm, Institut de Myologie, Centre de Recherche en Myologie, 75013 Paris, France; ^7^ Confocal Microscopy and Cellular Imaging Unit, Department of Genetic and Molecular Medicine, Hospital Sant Joan de Déu, 08950 Barcelona, Spain; ^8^ Institut de Recerca Sant Joan de Déu, Esplugues de Llobregat, 08950 Barcelona, Spain; ^9^ Statistics Department, Institut de Recerca Sant Joan de Déu, 08950 Barcelona, Spain; ^10^ Institut de Neurociències, Universitat Autònoma de Barcelona, 08193 Barcelona, Spain; ^11^ Department of Cell Biology, Physiology and Immunology, Universitat Autònoma de Barcelona, 08193 Barcelona, Spain

**Keywords:** Collagen VI-related dystrophies, Dominant-negative mutation, CRISPR/Cas9, Mouse model, Myopathy, Fibrosis, Automated image analysis

## Abstract

Collagen VI related dystrophies (COL6-RD) are congenital muscle diseases, typically inherited as an autosomal dominant trait. A frequent type of pathogenic variant involves glycine substitutions in the triple helical domain of collagen VI alpha chains, exerting a dominant-negative effect on the unaltered protein. Despite this, no prior animal model captured this mutation type. By using CRISPR/Cas9, we generated transgenic mice with the equivalent of the human *COL6A1* c.877 G>A; p. Gly293Arg pathogenic variant. We characterized their skeletal muscle phenotype over time, utilizing computer-aided tools applied to standardized parameters of muscle pathology and function. Knock-in mice exhibited early-onset reduced muscle weight, myopathic histology, increased fibrosis, reduced collagen VI expression, muscle weakness and impaired respiratory function. These features provide adequate outcome measures to assess therapeutic interventions. Different automated image analysis methods deployed here are able analyze thousands of features simultaneously, enhancing accuracy in describing muscle disease models. Overall, the *Col6a1* Ki Gly292Arg mouse model offers a robust platform to deepen our understanding of COL6-RD and advance its therapeutic landscape.

## Teaser

**Summary:** Generation and characterization of a mouse model comprising dominant-negative glycine substitutions within collagen VI alpha chains, a frequent cause of collagen-VI-related dystrophies.

## INTRODUCTION

Collagen VI-related dystrophies (COL6-RD) encompass a spectrum of ultra-rare myopathies of varying progression and severity from Ullrich congenital muscular dystrophy (UCMD) to Bethlem myopathy (BM) with intermediate phenotypes between them. COL6-RD clinical hallmarks include muscle weakness, distal joint hyperlaxity, proximal joints contractures and progressive respiratory insufficiency in a proportion of affected children. COL6-RD are orphan diseases lacking an effective therapeutical approach. The only treatment available is symptomatic and based on physical and respiratory therapy, surgery and ventilatory support (Natera de Benito et al., 2020).

Collagen VI is an extracellular matrix protein that is expressed in many tissues, including tendon, ligaments and skin, which are also affected in individuals with COL6-RD. Collagen VI is synthesized and secreted by tissue-specific mesenchymal cells, such as fibroadipogenic precursor cells, which reside between muscle fibers and are the major source of collagen VI in skeletal muscle (Zou et al., 2008). The three collagen-VI polypeptide chains (alpha 1, 2 and 3) form a heterotrimer, which then associates with another heterotrimer to form dimers and then tetramers, which are secreted into the extracellular matrix. In the extracellular space the tetramers associate to form microfibrils, with a characteristic beaded-filament aspect that, amongst other functions, mediates adhesion of the muscle fiber to the surrounding matrix (Cescon et al., 2015; Paco et al., 2015).

COL6-RD is caused by variants in any of the three major collagen VI genes (*COL6A1*, *COL6A2*, *COL6A3*). The majority (∼75%) of mutations are *de novo* dominant variants (Allamand et al., 2011; Lamandé and Bateman, 2018). Amongst those, glycine to arginine substitutions in the N-terminus of the triple helical collagen domain are common amongst individuals with intermediate and milder forms of COL6-RD. The mutant alpha chains carrying these glycine substitutions associate with the alpha chains encoded by the wild-type allele and are incorporated into tetramers that will be secreted into the extracellular matrix. The tetramers containing mutant chains interfere with the correct assembly and function of the tetramers – formed only by wild-type molecules – exerting a dominant negative effect, and impairing collagen VI microfibril formation and function. Common missense variants in the region of the triple helix are the ones affecting substitutions of glycine (G) to arginine residues at positions 284, 290 and 293 (G284R, G290R and G293R) in exons 9 or 10 of the *COL6A1* gene. The effect of these glycine substitutions has been characterized at the biochemical level in skin fibroblasts of patients diagnosed with COL6-RD, a cell model commonly used for diagnosis and pre-clinical research of COL6-RD (Butterfield et al., 2013; Jimenez-Mallebrera et al., 2006).

We and other groups have reported promising *in-vitro* results by using siRNAs, ASOs or CRISPR/Cas9 (Noguchi et al., 2014; López-Márquez et al., 2022; Brull et al., 2024) aimed at reducing the expression of the G284R and G293R alleles and, thus, their detrimental effect. These approaches have demonstrated significant silencing of the mutant allele, which was accompanied by increased in collagen VI matrix deposition, improved architecture of the collagen VI microfibrillar network and recovery of various subcellular alterations (Castroflorio et al., 2022).

Research in collagen-VI-deficient primary cells and in the existing animal models for recessive and dominant COL6-RD has identified various underlying molecular alterations, including fibrosis and extracellular matrix remodeling, impaired autophagy, mitochondrial dysfunction, muscle cell atrophy and intrinsic stem cell defects (Lamandé and Bateman, 2018). Several of those studies were conducted in fibroblasts and/or muscle biopsies from individuals diagnosed with COL6-RD, carrying one of those frequent glycine substitutions. However, there is no reported mouse model that represents this typical type of mutation. By using CRISPR/Cas9, we have generated a knock-in (Ki) mouse model that harbors the *Col6a1* c.874 G>A; p. G292R mutation, which mimics the human pathogenic variant *COL6A1* c.877 G>A; p. G293R. We use the term *Col6a1* Ki G292R mutant mice to refer to both the heterozygous and homozygous genotypes and the term wild type, heterozygous and homozygous to refer to Col6a1^+/+^, Col6a1^G292R/+^ and Col6a1^G292R/ G292R^ genotypes, respectively.

We used this model to investigate mutation-specific and general disease drivers and modifiers, derive useful read-outs to guide future pre-clinical studies and validate potential therapies. We systematically characterized this novel mouse model by deploying different qualitative and quantitative tools on standardized muscle pathology parameters. *Col6a1* Ki G292R mice show hallmarks of COL6-RD, such as fibrosis, partial collagen-VI deficiency and reduced limb strength, provide some new insights into the disease and, overall, constitute a valuable mouse model for COL6-RD.

## RESULTS

### Generation of *Col6a*1 knock-in mice by using CRISPR/Cas9

**Fig. 1.**
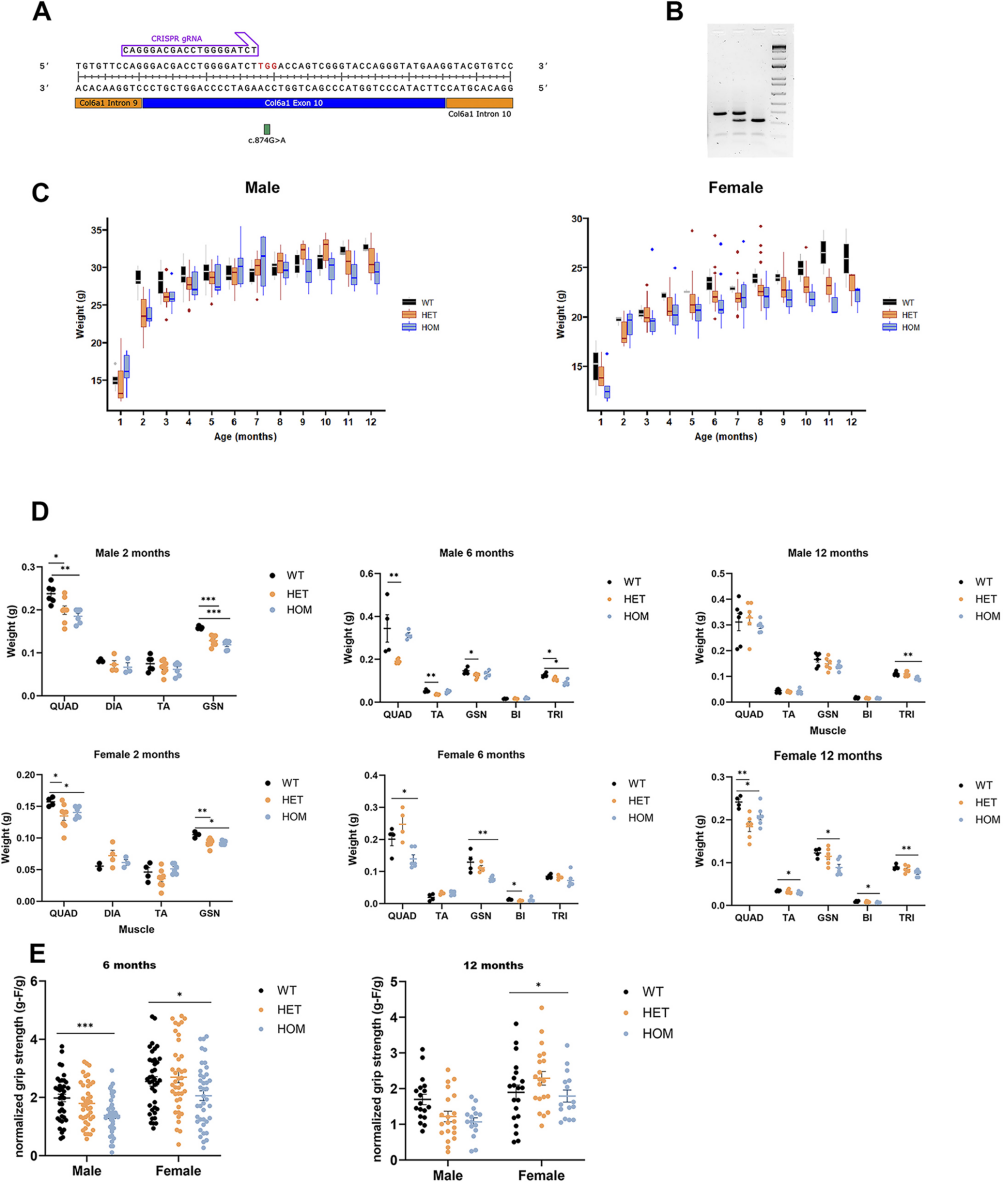
**Generation and overall characterization of *Col6a1* Ki G292R mice.** (A) Position of guide RNA (gRNA) used to generate *Col6a1* Ki mice relative to the position of the introduced mutation in the *Col6a1* gene. (B) Representative agarose gel electrophoresis of digestion fragments corresponding, from left to right, to wild-type (see two bands of 380 bp and 120 bp), heterozygous (see three bands of 380 bp, 307 bp and 120 bp) and homozygous (see two bands of 307 bp and 120 bp) genotypes. (C) Plotted is the progression of body weights in male and female mice over a 12-month period. A linear regression model was used (see Materials and Methods), median and interquartile ranges are represented). (D) Plotted is the weight of different muscles obtained from male (top) and female (bottom) mice at 2, 6 and 12 months of age. (E) Grip strength of the forelimbs was assessed separately for 6- and 12-month-old male and female mice and is provided as gram-force/gram of body weight (g-F/g). In D and E, means and SEM are represented. One-way ANOVA was used followed by Tukey's HSD as a post-hoc test to determine statistical significance; *n*=4-8 mice per sex and age. *P<0.05, **P<0.01, ***P<0.001, ****P<0.0001. WT, wild-type (Col6a1^+/+^); HET, heterozygous (Col6a1^G292R/+^); HOM, homozygous (Col6a1^G292R/ G292R^).

We designed a guide RNA (gRNA) targeting exon 10 by using the Breaking-Cas tool (Oliveros et al., 2016) and a 200-bp single-stranded oligodeoxynucleotide (ssODN) template that contained the desired muted base (c.874 G>A) (Fig. 1A). Both gRNA and template were microinjected with SpCas9 into C57BL6/J zygotes, and implanted in pseudopregnant females. Edited founders were identified by PCR amplification (Table S1) and those carrying the desired alleles, were crossed five generations with wild-type C57BL6/6J to eliminate possible unwanted off-targets. Heterozygous mice were re-sequenced and crossed with C57BL6/6J wild-type animals to generate the BL/6J- Col6A1em1(c.874G>A) ^Sal^ mouse colony which we refer to as *Col6a1* Ki G292R.

### *Col6a1* Ki G292R mice show reduced growth, muscle atrophy and weakness

Mutant mice moved and bred normally, had a normal life span, and were not overtly different from wild-type mice. We obtained the expected number of mice per genotype in offspring according to Mendelian inheritance and a comparable number of animals of each sex. Regarding the growth of the mice in terms of body weight, we observed differences between genotypes (and sexes) that we explored using piecewise and linear regression models for males and females separately between the ages of 1 month and 12 months. Based on the available data, we observed that body weight progression followed a two-phase pattern: an initial phase of rapid growth, followed by a second phase of slower growth.

We applied a linear regression model with a segmented relationship that estimated the breakpoint between the two phases to occur at approximately 2.2 months (95% CI 2.01-2.47). Then, we analyzed each period separately and studied how age, sex and genotype relates to gaining body weight (Fig. 1C). The values of the resulting coefficients and adjusted *P* values were included in Table S2. In male mice, the wild-type group grew more rapidly in the first phase than heterozygous and homozygous mice (adjusted *P*-value <0.05 in both cases represented by the coefficient Age×Genotype HET or HOM in Table S2), indicating that they reached their adult weight faster. In the second phase we also obtained significant results indicating that heterozygous mice gain body weight starting from a lower baseline (corrected *P*-value <0.05, represented by the coefficient Age×Genotype HET in Table S2). However, all mice eventually reached similar weight by 12 months. In female mice, the analysis did not support growth rate differences between genotypes (Table S2).

We analyzed the weight and morphology using Hematoxilin and Eosin staining (H&E) of the liver, kidney, brain, lung, intestine and spleen from 6- and 12-month-old mice separately in males and females to reveal any potential subjacent non-skeletal pathology. However, no major changes regarding the normalized weight of the organs or tissue morphology related to the genetic modification were observed (data not shown). Regarding the weight of individual muscles, those from *Col6a1* Ki G292R mice weighed less than the muscles of their wild-type littermates and remained so up to the age of 12 months (Fig. 1D).

We then tested the strength of forelimbs of individual mice to evaluate whether presence of the pathogenic variant also affects the ability to grip and generate strength. Despite the variability in measured strength between individuals, we determined a significant reduction (*P*<0.05 in females, *P*<0.001 in males) in strength between wild-type and homozygous mice aged 6- or 12-months old, which was more pronounced in males than females (Fig. 1E). On average, forelimb strength of heterozygous mice was reduced compared to that of the wild-type group, but differences were not statistically significant.

### Respiratory function evolves differently in *Col6a1* Ki G292R mice compared to wild-type mice

**Fig. 2.**
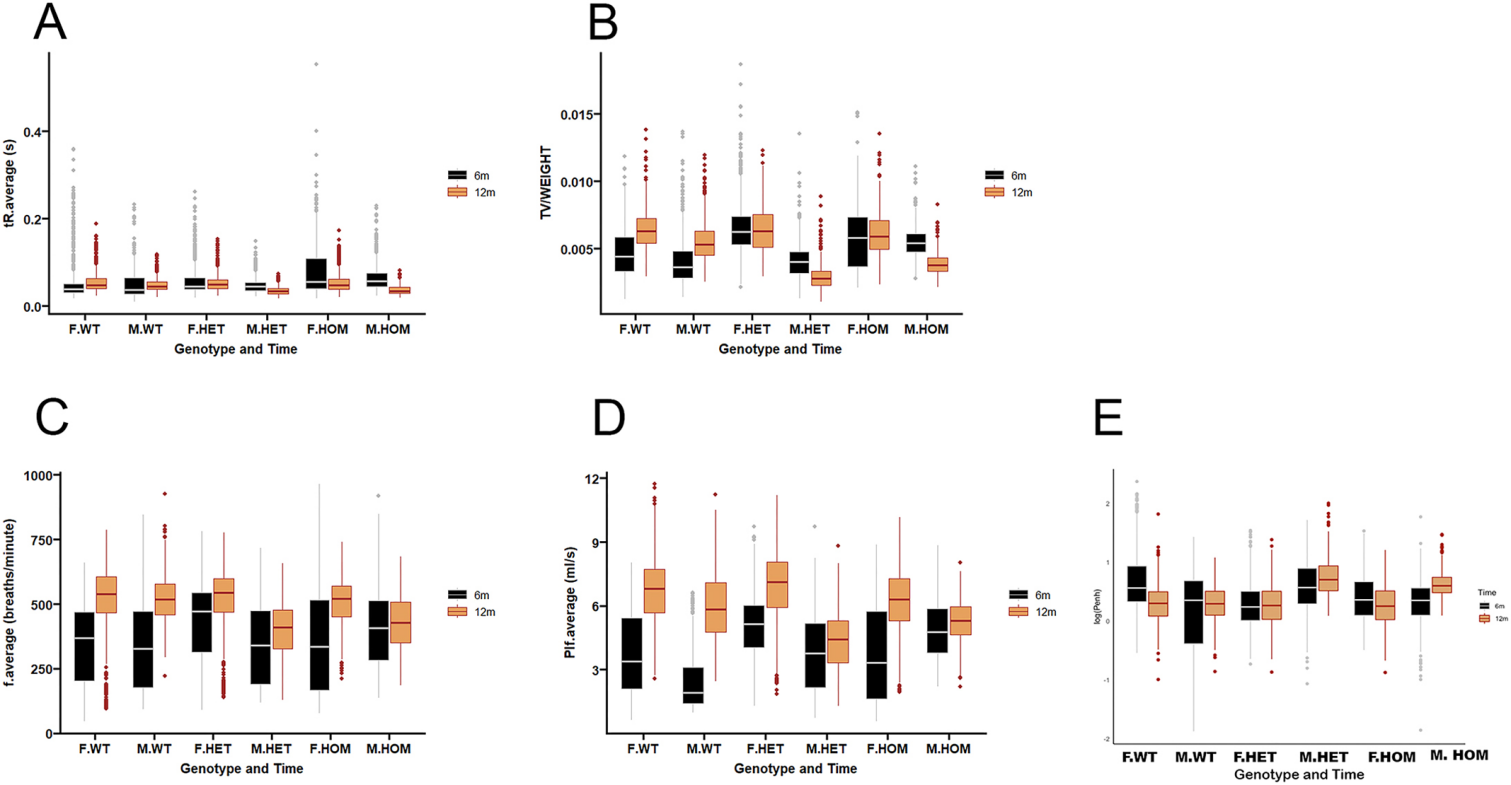
**Respiratory function assessed by whole-body plethysmography.** (A-E) Distribution of respiratory-function parameters by sex and genotype at 6 and 12 months. Analyzed were average relaxation time (tR) (A), tidal volume/weight of the animal (TV/WEIGHT) (B), average respiratory rate (f.average) (C), average peak inspiratory flow (Pif.average) (D) and logarithmic pause and enhanced pause [log(Penh)] (E). See Materials and Methods for details of the statistical analysis and Table S2 for adjusted *P*-value details. F.WT, female wild-type; M.WT, male wild-type; F.HET, female heterozygous; M.HET, male heterozygous; F.HOM, female homozygous; M.HOM, male homozygous.

We investigated respiratory function in *Col6a1* Ki G292R mice by using whole-body plethysmography (WBP), a non-invasive technique that allows recording several indicators of respiratory function. We recorded data for male and female wild-type, heterozygous and homozygous mice separately at two timepoints, i.e. in mice aged 6 and 12 months. We applied a linear mixed model to investigate the effect of three factors: genotype, sex and moment in time, as well as their interactions. The values of the resulting coefficients and adjusted *P*-values for each parameter are listed in Table S3. Some parameters, i.e. end inspiratory pause and end expiratory pause, were excluded because of the presence of outliers (data not shown). For data of the other parameters – relaxation time, volume of air inspired and expired with each passive breath (tidal volume) corrected by weight; respiratory rate, peak inspiratory flow, and pause and enhanced pause (Penh) – see Fig. 2.

At 6 months, the average relaxation time was significantly longer (*P*<0.001) in homozygous mice relative to wild-type mice. When we analyzed the changes in the different respiratory parameters in mice aged 6 and 12 months. We observed significant (*P*<0.001) increases in tidal volume, respiratory rate and peak inspiratory flow in wild-type mice of either sex. However, these increases were not observed for heterozygous or homozygous mice. The Penh index decreased (*P*<0.001) in wild-type mice, but increased in heterozygous or homozygous male mice (*P*<0.001) over time. In summary, WBP showed that, compared to wild-type littermates, respiratory function evolves differently in *Col6a1* Ki mice, and this difference is more substantial in male compared to female mice.

### *Col6a1* Ki G292R mice show early myopathic changes, including increased fibrosis in various muscles such as limb muscles and the diaphragm

**Fig. 3.**
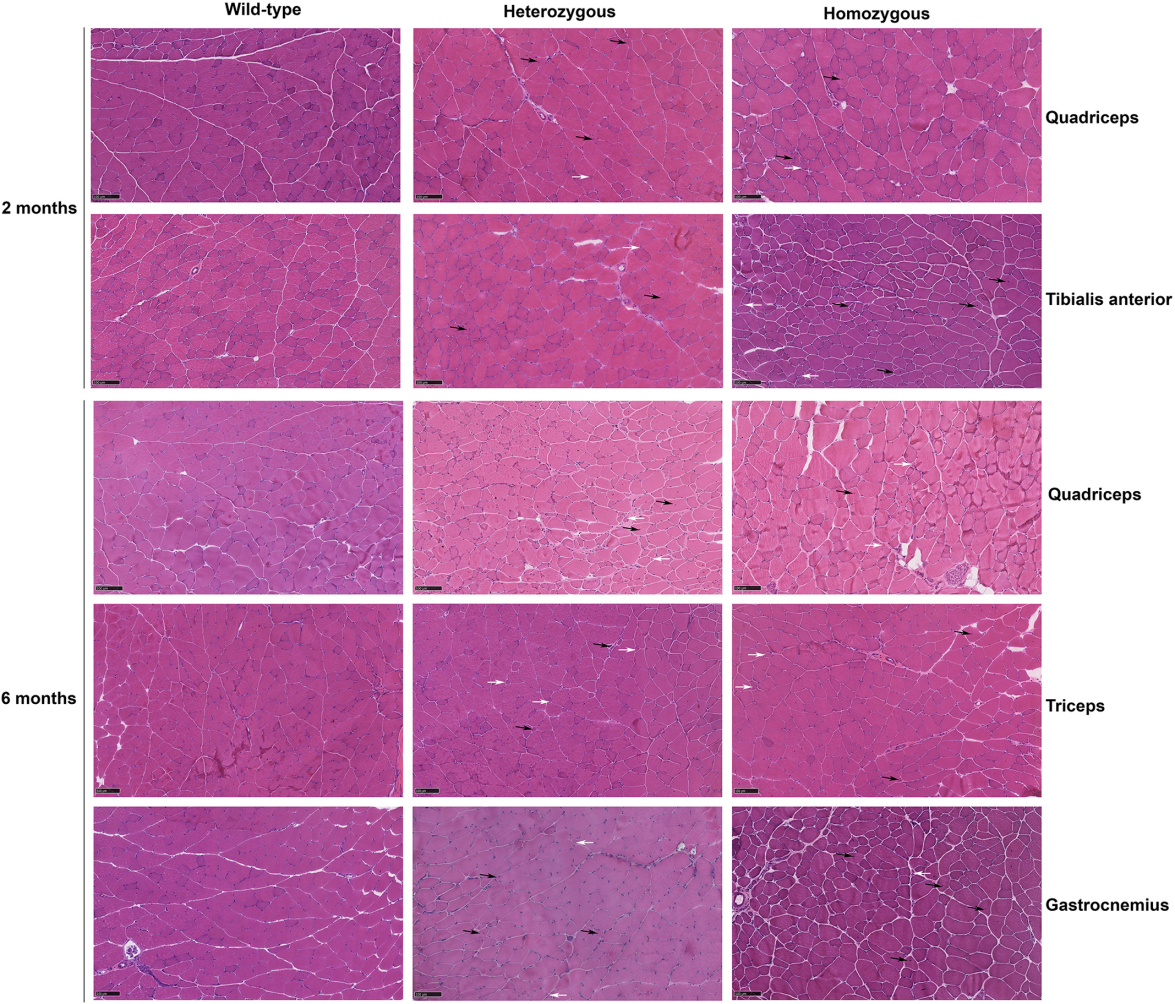
**Histological analysis.** Images of frozen sections of different muscles as indicated were stained with Hematoxilin and Eosin (H&E). Myopathic features include fiber size variability with small atrophic fibers (white arrows) and increased internal nuclei (black arrows). Scale bars: 100 µm.

We qualitatively evaluated the overall skeletal muscle pathology in different muscles (quadriceps, tibialis anterior, gastrocnemius, soleus, biceps, triceps, diaphragm), of all three genotypes at age 2, 6 and 12 months in male and female mice by using H&E staining. First, two examiners, who were unaware of the experimental conditions, conducted a qualitative assessment of the histological preparations; then, one of them, with more experience in muscle pathology, analyzed the preparations in more detail. We observed mild pathological changes at 2 months in heterozygous and homozygous mice, in the different limb muscles examined and the diaphragm, i.e. the presence of internal nuclei, variation in fiber size and roundness of muscle fibers. In mice at 6 months of age, we observed a similar degree of pathology in all muscles, including triceps and biceps. In some cases, the pathology appeared more substantial in the homozygous compared with heterozygous mice, but this was not consistent and depended on the individual mouse and the muscle (Fig. 3). In mice at 12 months, the pathological changes persisted but did not seem to worsen with age (Fig. S1). Differences in the diaphragm between heterozygous and homozygous and wild-type mice were more apparent compared with younger mice, although the pathology of the diaphragm is difficult to interpret because of the technical difficulty in orienting the muscle fibers and in avoiding artifacts. In summary, this qualitative morphological analysis revealed mild pathological changes from 2 months of age in all limb muscles and in the diaphragm that were compatible with a myopathic process.

To quantitate and statistically evaluate muscle pathology, we applied an automated purpose-built pipeline on whole-section reconstructions of gastrocnemius, quadriceps, tibialis anterior and triceps from 6-month-old mice (see Materials and Methods) analyzed separately by sex. This allowed us to obtain many fibers for each muscle (ranging from 893 to >9000). We focused on two parameters. First, the minimum Feret diameter, a robust measurement of muscle fiber size because it avoids errors due to the orientation of the sectioning angle and is widely used to describe the variation in muscle fiber diameter in animal models of muscular dystrophy. Second, the presence and number of internal nuclei, an indicator of muscle degeneration and regeneration.

Regarding the Feret diameter, we analyzed differences between genotypes by using standard statistical tests, i.e. Kruskal–Wallis test to compare the values obtained from these three groups, followed by Mann–Whitney *U*-test with Bonferroni−Holm correction for multiple testing as post-hoc analysis. We observed significant differences in all comparisons except for female gastrocnemius (Table S4).

However, because of the very high number of fibers analyzed, we expected to obtain statistically significant differences. For this reason, we calculated the effect size (r) for each comparison, which qualifies the magnitude of a difference between groups. A statistically significant result with a small r value might not be practically meaningful, while a large r value indicates a more substantial contribution to the differences between groups. We applied a threshold of 0.1 so that r<0.1 is not considered relevant and r>0.1 is considered relevant (r values for each comparison are provided in Table S4).

The application of r value correction revealed substantial differences in the minimum Feret diameter between genotypes in different muscles, although the direction of the change was not constant (Fig. S2). In gastrocnemius and triceps, fibers from homozygous male mice were larger than fibers from heterozygous and wild-type mice, whereas, in quadriceps, Feret diameter values were smaller in homozygous and heterozygous mice than in wild-type mice. In the tibialis anterior, homozygous mice fibers were smaller than fibers from heterozygous and wild-type mice. In female mice, we observed that in, quadriceps, the minimum Feret diameter was reduced in heterozygous mice, whereas, in tibialis anterior and triceps, it was increased relative to wild-type mice. We also calculated the coefficient of variation and its confidence interval (95% CI) as another indicator of fiber size variability (Table S5).

For the number of internal nuclei, we looked at the percent distribution of fibers with zero or more internal nuclei. There were significant and relevant differences between genotypes only in the tibialis anterior of male mice (adjusted *P*-value <0.001, r>0.1). In this case, in heterozygous mice there was a significant and relevant lower proportion of fibers with 0 internal nuclei (87. 3%) than in wild-type mice (96.1%).

**Fig. 4.**
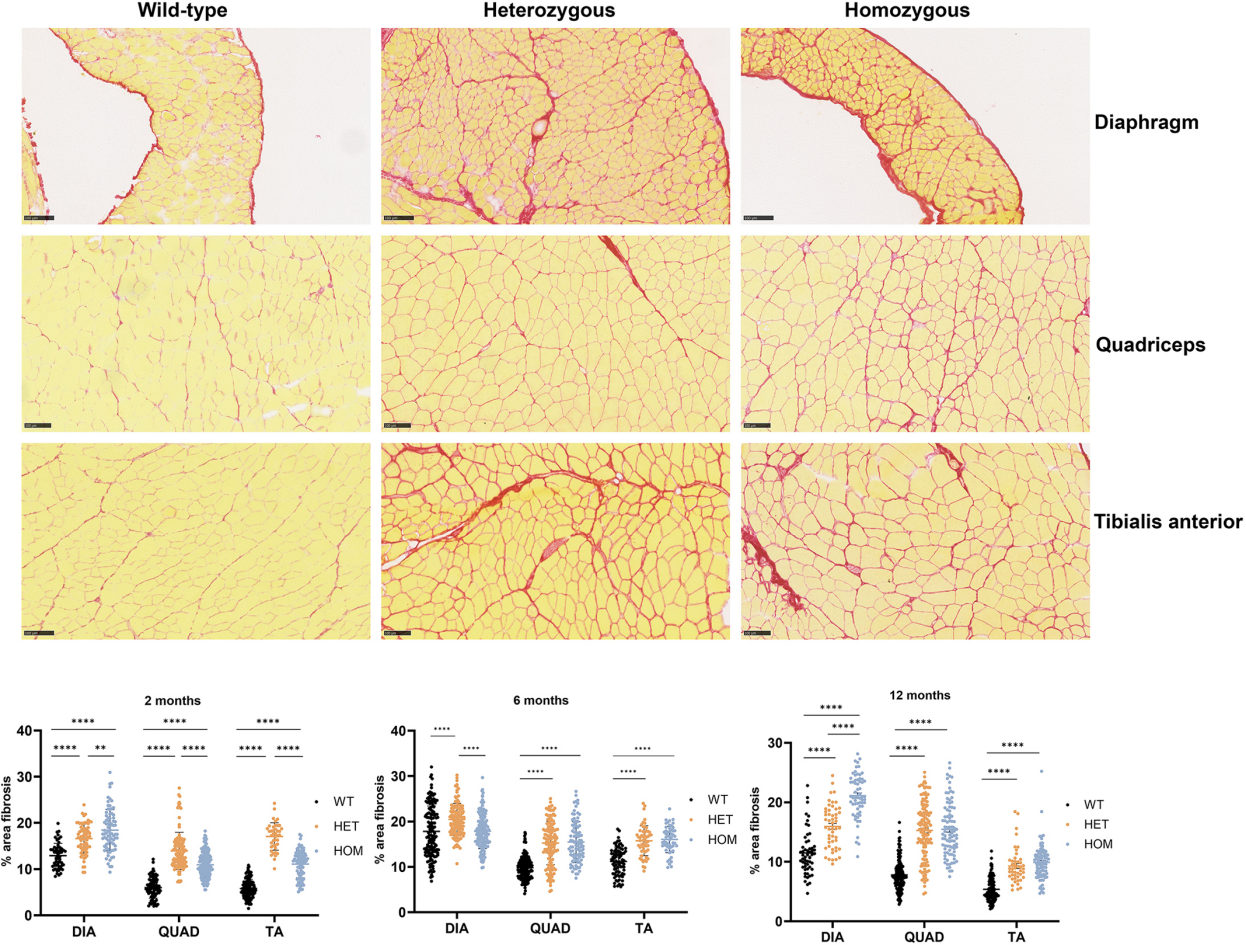
**Fibrosis analysis.** Top panel: Images of diaphragm, quadriceps and tibialis anterior sections from mice as indicated, stained with Picro Sirius Red reveal collagen deposition as an indicator of fibrosis. Scale bars: 100 µm. Bottom panel: Violin plots showing the quantification of the percentage (%) area of fibrosis in the diaphragm (DIA), quadriceps (QUAD) or tibialis anterior (TA) of wild-type (WT), heterozygous (HET) and homozygous (HOM) mice aged 2, 6 or 12 months. **P<0.1, ****P<0.0001.

To determine the presence and extent of endomysial fibrosis, we stained sections using Picro Sirius Red and measured the percentage of the area occupied by collagen fibrils. We then quantitatively analyzed the diaphragm, quadriceps and tibialis anterior in 2-, 6- and 12-month-old mice, by applying an automatic image analysis pipeline that we programmed with FIJI-ImageJ Software. A significant increase in collagen deposition was observed in 2-month-old mice within the three muscles of heterozygous and homozygous mice relative to wild-type littermates (Fig. 4). The same trend was observed in 6- and 12-month-old mice, including triceps − which were not collected from mice aged 2 months. In some cases, there were also significant differences between heterozygous and homozygous mice, e.g. in the diaphragm from 2-, 6- and 12-month-old mice, Fig. 4). In summary, we observed pathological fibrosis in all muscles tested in knock-in mice.

### Mouse diaphragm shows the highest expression of *Col6a1*, and expression of *Col6a3*, *Col6a5* and *Col6a6* is unchanged in *Col6a1* Ki mice

**Fig. 5.**
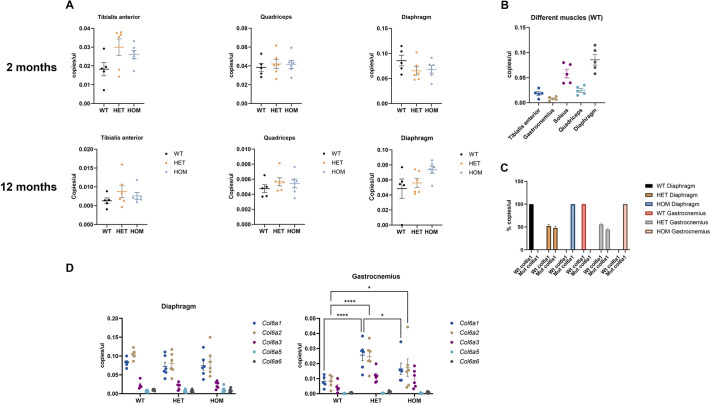
**Expression of Col6a transcripts.** (A) *Col6a1* expression in tibialis anterior, quadriceps and diaphragm of wild-type (WT), heterozygous (HET) and homozygous (HOM) 2-month-old (top) and 12-month-old (bottom) mice (*n*=5 (WT), 6 (HET), 6 (HOM). (B) Comparison of total *Col6a1* levels in different muscles in WT mice at 2 months (*n*=5). (C) Abundance (copies/µl) of *Col6a1* wild-type (Wt) and mutant (Mut) *Col6a1* transcripts in diaphragm and gastrocnemius in 2-month-old wild-type (WT; *n*=5), heterozygous (HET; *n*=6) or homozygous (HOM; *n*=6) mice. (D) Expression of *Col6a1*, *Col6a2*, *Col6a3*, *Col6a5* and *Col6a6* in diaphragm and gastrocnemius. Two-way ANOVA was performed to compare the different groups, followed by Tukey's multiple comparison test for two-way comparisons between genotypes. Expression levels are reported as copies/μl and normalized to *Gapdh*. Data from two experiments are represented as mean±s.e.m. *P<0.05, **P<0.01, ***P<0.0005, ****P<0.0001. WT, wild-type (Col6a1^+/+^); HET, heterozygous (Col6a1^G292R/+^); HOM, homozygous (Col6a1^G292R/ G292R^).

We analyzed the expression of *Col6a* transcripts by digital droplet PCR in different muscles of 2- and 12-month-old mice (Fig. 5). We either measured total *Col6a1*, *Col6a2*, *Col6a3*, *Col6a4* and *Col6a5* mRNAs levels or used allele-specific probes to determine the expression of *Col6a1*-expressing wild-type and mutant alleles, to investigate whether any deviation from the expected 50% expression of each allele occurred. We did not observe significant differences between males and females in total *Col6a1* expression levels, so data were pooled (Fig. 5). In some muscles, *Col6a1* transcript levels were higher in heterozygous and/or homozygous mice than in wild-type mice; although, in most cases, those differences were not significant (Fig. 5A and D). When we compared *Col6a1* copy number/µl of across the different muscles tested in wild-type mice, we found that diaphragm was the tissue expressing the highest levels, followed by soleus, tibialis anterior, quadriceps and gastrocnemius (Fig. 5B). The same results were observed in mice aged 12 months (data not shown).

By using allele-specific probes, we determined the ratio of wild-type (G residue at position 872) versus mutant (A residue at position 872) *Col6a1* allele. The percentage of each allele in heterozygous mice only slightly deviated from the expected 50% ratio (Fig. 5C).

Expression of *Col6a5* and *Col6a6* has been described in a variety of mouse tissues (Fitzgerald et al., 2008). We assessed the expression levels of *Col6a2*, *Col6a3*, *Col6a5* and *Col6a5* in two representative muscles (gastrocnemius and diaphragm) in 2-month-old mice. Expression of *Col6a1* and *Col6a2* transcripts was much higher than that of *Col6a3*, *Col6a5* and *Col6a6* transcripts in both muscles tested. *Col6a2* was upregulated in heterozygous and homozygous mice in gastrocnemius, but in wild-type and knock-in mice no differences were detected expression levels of *Col6a3*, *Col6a5* or *Col6a6* (Fig. 5D).

### Levels of collagen VI are reduced in *Col6a1* Ki muscles and diaphragm in mice at different ages

**Fig. 6.**
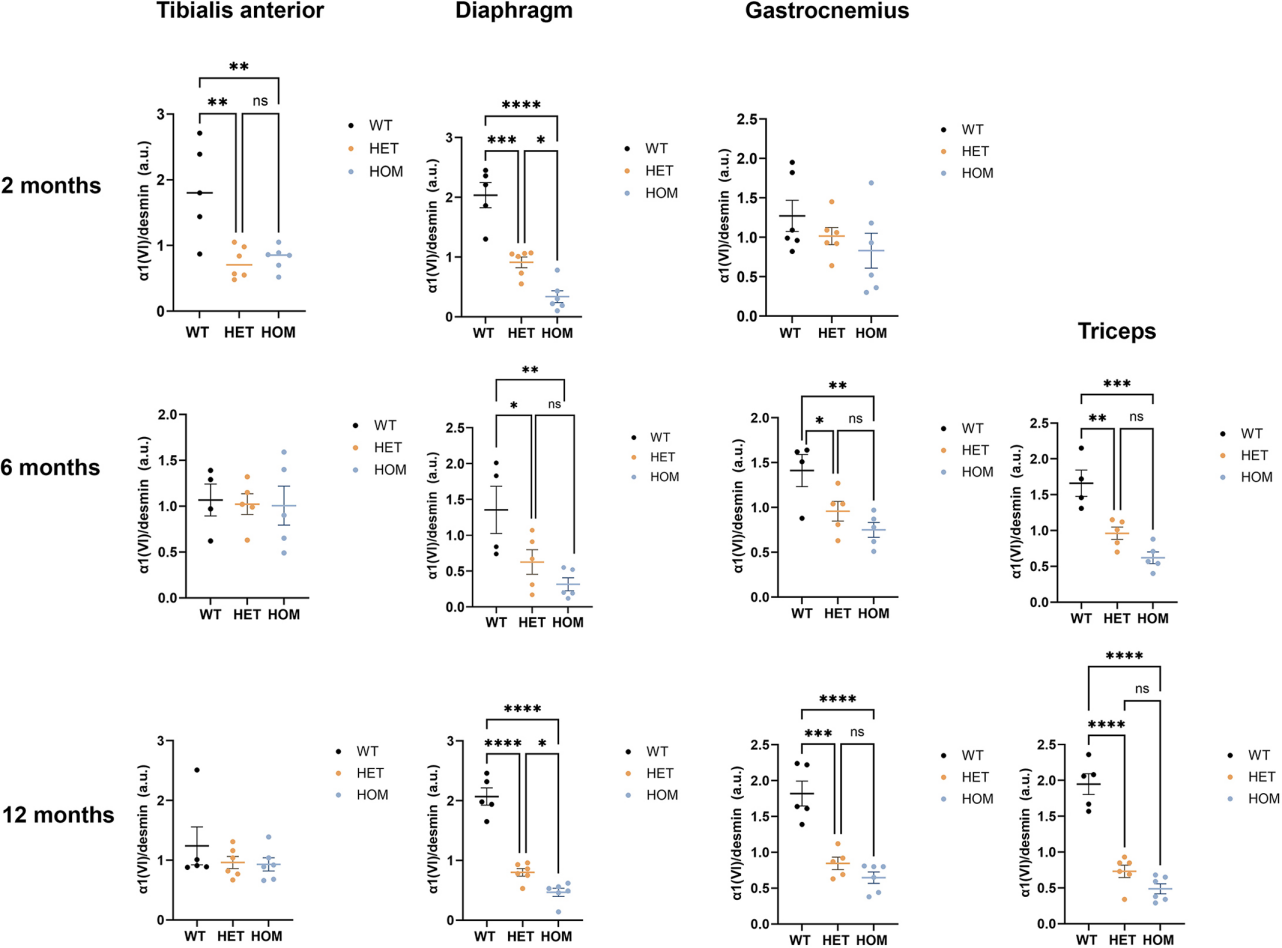
**Quantitative analysis of immunoblotting for collagen alpha 1 (VI).** Bars graphs showing the relative abundance of collagen alpha 1 (VI) in triceps, diaphragm, gastrocnemius and tibialis anterior muscles of wild-type, heterozygous and homozygous mice at different ages (*n*=3-6 mice per genotype per stage). Col6a1 was detected with a polyclonal antibody against collagen type VI (70R-CR009x). Data were analyzed using one-way ANOVA followed by Tukey's multiple comparison test. Data are the mean±s.e.m. *P<0.05, **P<0.011, ***P<0.001, ****P<0.0001. WT, wild-type (Col6a1^+/+^); HET, heterozygous (Col6a1^G292R/+^); HOM, homozygous (Col6a1^G292R/ G292R^).

To detect and semi-quantify collagen VI alpha chains, we used SDS-PAGE and western blotting to analyze the presence and relatively abundance of the collagen alpha 1 (VI) polypeptide in total protein extracts of different muscles obtained from mice aged 2, 6 and 12 months. First, we compared males and females but found no significant differences. Second, data from male and female mice were analyzed together. We observed a significant reduction in the relative levels of collagen alpha 1 (VI) in all the muscles tested (triceps, diaphragm, gastrocnemius and tibialis anterior) at least at one of the three ages analyzed (Fig. 6, Fig. S3).

**Fig. 7.**
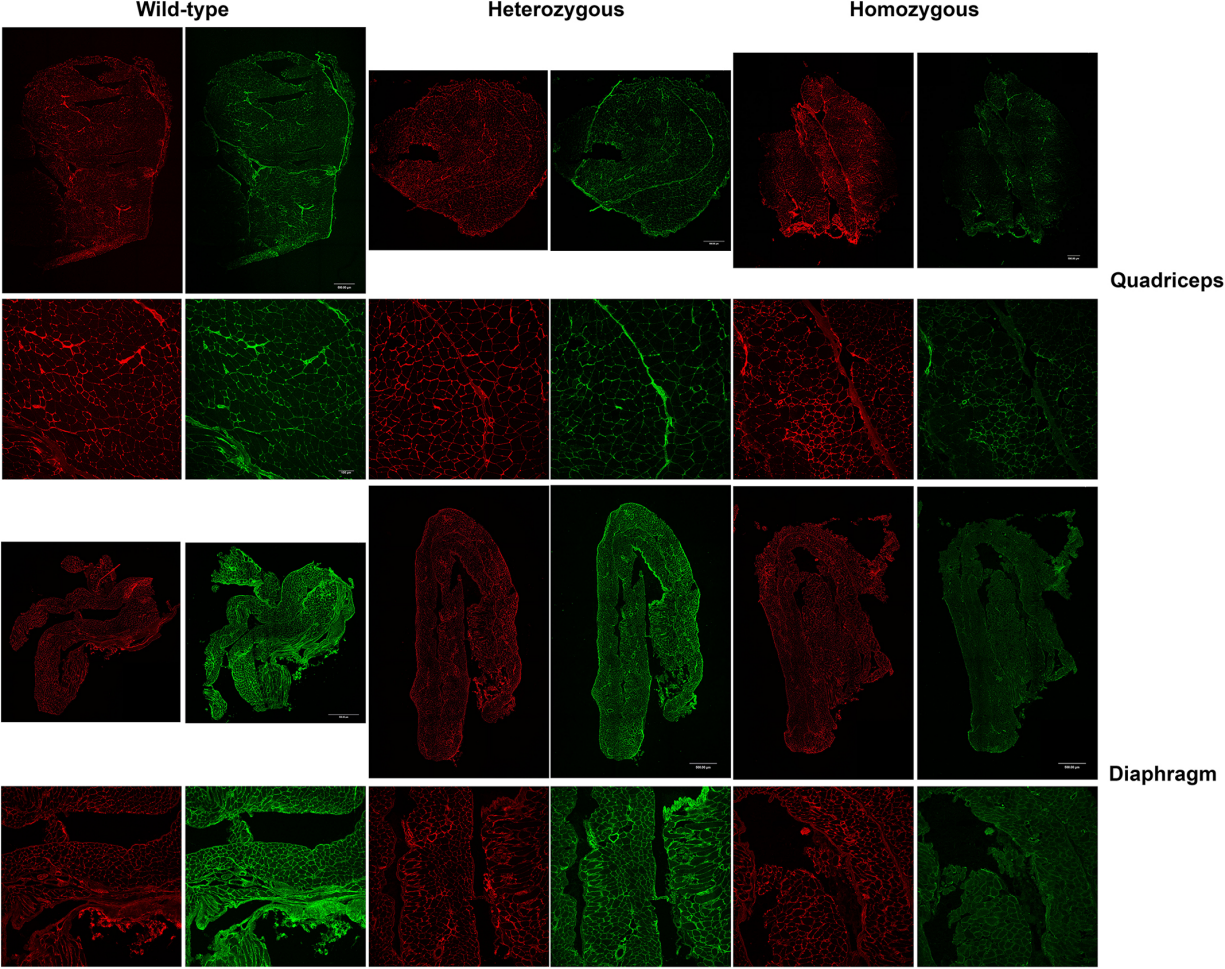
**Immunofluorescence staining for collagen VI and perlecan.** Confocal microscopy images of quadriceps and diaphragm muscle sections from 12-month-old mice stained for collagen VI (green) or perlecan (red). Top two images for each muscle represents whole section mosaics (scale bars: 500 µm); bottom two images for each muscle show a representative area from the respective top image the respective top image. Scale bars: 100 µm. Wild-type, Col6a1^+/+^; Heterozygous, Col6a1^G292R/+^; Homozygous, Col6a1^G292R/ G292R^. Corresponding merged images are shown in Fig. S4.

Muscle cryosections from 12-month-old mice were double-immunostained for collagen VI and perlecan, with perlecan being used as control for basal lamina integrity in human biopsies. A minimum of two slides (four sections) per muscle and mouse were processed from two separate positions of the muscle block. Confocal microscopy images of whole-section mosaics were captured, muscle fibers segmented and fluorescence intensity around each muscle fiber was automatically measured. This automation allowed us to quantify a very high number of muscle fibers per muscle. Since an initial analysis of collagen VI fluorescence intensity in some muscle samples obtained from male and female mice did not reveal significant differences, analysis was performed irrespectively of sex. We compared for each genotype the ratio between collagen VI and perlecan. In all muscles analyzed (biceps, diaphragm, quadriceps, gastrocnemius, soleus and triceps), except tibialis anterior, the ratio between collagen VI and perlecan was significantly decreased in heterozygotes and homozygous mice relative to the wild-type littermates (Fig. 7, Fig. S4). The greatest reduction in terms of mean fluorescence intensity and regarding the extent of the statistical significance was observed in diaphragm, quadriceps and soleus (Fig. S5, Table S6).

## DISCUSSION

In this work, we describe a new mouse model of collagen VI related dystrophies (COL6-RD), for which CRISPR-Cas9 technology was used to introduce a missense mutation in exon 10 of the mouse C*ol6a1* gene. This mutation affects the amino acid G292 within the N-terminus of the collagen alpha-1 (VI) chain – the equivalent to amino acid G293 within the human protein. The G293R transition, as well as other equivalent transitions in that region of the alpha 1 chain, are a common type of variant in affected individuals; therefore, this novel mouse model is relevant for COL6-RD research. To our knowledge, this is the first knock-in mouse model of COL6-RD with this type of pathogenic variant, which is associated with the intermediate and milder forms of COL6-RD.

We provide evidence of pathological and functional alterations in mutant mice compatible with a myopathic process. This includes reduced muscle mass, excess deposition of endomysial collagens, as an indication of fibrosis, from an early age, a partial deficit in collagen VI protein levels and localization at the basal lamina, reduced strength and impaired respiratory function. These features are also observed in individuals diagnosed with COL6_RD, carrying the *COL6A1* G293R variant (Paco et al., 2012, 2013; Butterfield et al., 2013).

Other mouse models for dominant-negative mutations include mice with deletion of exon 16 of *Col6a3* (Pan et al., 2014), deletion of exon 5 of *Col6a2* (de Greef et al., 2016) or humanized mice carrying the variant *COL6A1* 930+189C>T (Bolduc et al., 2024 preprint). In general − in terms of reduced muscle mass and limb strength, and regarding mild myopathic features, including interstitial fibrosis (Pan et al., 2014) − these models show similar features to the *Col6a1* knock-in mice reported in our study here.

Pulmonary function is a relevant disease outcome measure in the context of COL6-RD. In affected individuals, this is regularly assessed with the measurement of forced vital capacity (FVC). In individuals with the intermediate and mild forms of COL6-RD, associated with the *COL6A1* het. c. 877 G>A variant, the predicted FVC is, on average, about 60% and 75% of the predicted value, and it has been estimated that, by the age of 20 years, 30% of individuals classified as having intermediate Ullrich congenital muscular dystrophy were using nocturnal ventilation in the form of bilevel positive airway pressure (BiPAP) (Natera de Benito et al., 2020). WBP is a very valuable experimental approximation to evaluate respiratory function in animal models. Analysis of pulmonary function has not been reported in the other mouse models of dominant collagen VI mutations, but has been reported in models of other congenital muscular dystrophies. For example, in laminin-2-deficient *dy^2J^* mice treated with an anti-apoptotic drug, the respiratory rate measured by WBP was a sensitive monitoring outcome measure (Yu et al., 2013). Similarly, we believe that the changes in various respiratory parameters we found over time in *Col6a1* G292R knock-in mice represent valuable pre-clinical physiological readouts to evaluate the effect of therapeutic interventions in this mouse model. Several challenges could be investigated to exacerbate this basal respiratory phenotype, such as exercise and hypoxia (i.e. oxygen deficiency in the tissue) and/or hypercapnia (i.e. high levels of carbon dioxide in the blood) (Hernández Rodríguez et al., 2025). When we analyzed the expression levels of the three main collagen VI genes (*Col6a1*, *Col6a2* and *Col6a3*), we observed increased expression of the *Col6a1* transcript in some muscles of our knock-in mice. We also observed this increase when we analyzed the RNA from fibroblasts of individuals carrying the equivalent *COL6A1* variant (A.L.-M., personal observation). Over-deposition of *Col6a5* and *Col6a6* chains into the extracellular matrix by immunofluorescence has been described in the mouse model lacking exon 16 of *Col6a1* (Pan et al., 2014). However, we did not detect changes in these two *Col6a* chains at the transcriptional level, which suggests that the overexpression of *Col6a5* and *Col6a6* may be regulated at the post-translational level.

By using immunoblotting, we evidenced a significant and consistent reduction in the relative levels of the *Col6a1* chain in different muscles in knock-in mice. Differences in the results obtained at the transcript level may be explained by the reduced stability or solubility of the protein chain that is not reflected in transcript levels. Although immunoblotting is not a quantitative technique it is, nonetheless, a useful tool to assess changes in collagen VI. The partial reduction in collagen VI intensity around muscle fibers by immunofluorescence and confocal microscopy is also a relevant finding because it is comparable to what it is usually found in COL6-RD affected individuals with dominant variants, including the *COL6A1* c.877 G>A nucleotide transition.

Although in humans the *COL6A1* c. 877 G>A variant is not homozygous, here we analyzed the heterozygous and the homozygous *Col6a1* G292R genotypes in parallel. All alterations were present in the heterozygous state, although, more aggravated in homozygous mice (for example the decrease in grip strength).

One of the aims of this study was to investigate image analysis pipelines to acquire and extract information from large datasets of morphology or intensity measurements. In the past, we have also applied quantitative image analysis to study other muscle conditions, such as Duchenne muscular dystrophy (Codina et al., 2023). We found that muscle fiber segmentation is a key step in this process, which can be automated by using existing tools, such as Cellpose3.0, but can be improved by manual curation.

Interestingly, we found that some morphological alterations, such as increased numbers of internal myonuclei apparent under the microscope when examining a relatively low number of muscles and optical fields, were not confirmed by the statistical analysis of hundreds or thousands of muscle fibers after size-effect correction. COL6-RD is not characterized by muscle fiber degeneration or regeneration so, perhaps, it is not surprising that internal nuclei were not confirmed to be the differential parameter between genotypes. In contrast, we and others have described the presence of atrophic fibers in COL6-RD human muscle biopsies at microscopic and ultrastructural level (Paco et al., 2012).

The observed morphological alterations could, nonetheless, be biologically relevant, although subtle. In any case, it highlights the importance of conducting extensive and quantitative analysis in disease models, particularly when they are used as a platform for therapeutic pre-clinical research. One way to increase the representativeness of the image analysis would be to collect the same high number of fibers, but from a greater number of mice, allowing individual variability to be incorporated into the statistical analysis.

One of the most-robust pathological findings was fibrosis observed from early age in heterozygous and homozygous *Col6a1* Ki mice in all muscles analyzed. Fibrosis has also been described in other COL6-RD mouse models and in other murine models of congenital muscular dystrophy (Pan et al., 2014; Mohassel et al., 2025). Fibrosis is also a frequent finding in muscle biopsies from individuals with COL6-RD. Extracellular matrix remodeling and fibrosis is also a common finding of various transcriptomic studies of collagen-VI-deficient muscle and fibroblasts (Paco et al., 2013). Recent studies have shown dysregulation of the TGFβ pathway leading to fibrosis and impaired regeneration after muscle injury in *Col6a2* knock-out mice as an early disease driver (Mohassel et al., 2025), and we plan to analyze these phenomena in the *Col6a1* Ki Gly292Arg mice.

It is worth mentioning that we did not consider generating a humanized model because the nucleotide transition of interest is in an exon, with the nucleotide position and corresponding amino acid being highly conserved between human and mice, and because of simplicity and cost-effectiveness. Furthermore, considering the high degree of homology between human and mouse counterparts in the coding region of the *COL6A1* gene, the sequences of nucleic acid-based therapeutics, such as antisense oligonucleotides, would be highly similar. Thus, the fact that it is not a humanized model does not preclude its value as pre-clinical model.

In summary, the *Col6a1* G292R mice generated by CRISPR/Cas9 represent the first knock-in model for dominant glycine substitutions in the triple-helical domain of collagen VI genes, which are a common type of genetic variant in individuals affected with intermediate and mild forms of COL6-RD. We have quantitatively determined – by using novel methods – several useful molecular, biochemical and functional outcome measures that are relevant to the human pathology. This model is, therefore, a valuable contribution to better understand the mechanisms of the dominant forms of COL6-RD, and to evaluate genetic and non-genetic therapeutic strategies.

## MATERIALS AND METHODS

### Generation of *Col6a1* knock-in mice by using CRISPR/Cas9, genotyping and mouse husbandry

For developing the *Col6a1* (c.874 G>A p.292G>R) mutant mouse model, a *Col6a1*-sgRNA1 – i.e. 5′-CAGGGACGACCTGGGGATCT-3′ − targeting the exon 10 was predicted using the Breaking-Cas gRNA designer tool (Oliveros et al., 2016). The crRNA and tracrRNA (Integrated DNA Technologies) used to obtain the mature *Col6A1*sgRNA were annealed by mixing them in equimolar amounts, and heating and cooling the mixture to allow hybridization. A designed 200 bp single-stranded oligodeoxynucleotide (ssODN) (*Col6a1*ssODN) contained the muted base (c.874 G>A) that mimics the human variant c.877 G>A (rs398123643:G/R) were chemically synthesized by Integrated DNA Technologies (IDT; IA, USA) (Table S1). A mixture containing the sgRNA (20 ng/μl), 30 ng/μl of recombinant Cas9 protein (IDT) and 10 ng/μl of the ssODN were microinjected into C57BL6/J zygotes at the Transgenic Facility of the University of Salamanca. Edited founders were identified by PCR amplification (Taq polymerase, NZYtech, Lisbon, Portugal) with primers flanking the edited region (Table S1). PCR products were directly sequenced or subcloned into pBlueScript (Stratagene) followed by Sanger sequencing. Selected founders, carrying the desired alleles, were crossed five generations with wild-type C57BL6/6J to eliminate possible unwanted off-targets. Heterozygous mice were re-sequenced and crossed with C57BL6/6J wild-type animals to generate the *BL/6J- Col6a1^em1Sal^* mouse colony.

For routine genotyping from ear samples, we used the Accustart II Mouse Genotyping Kit, (QuantaBio, MA, USA). DNA was amplified using a pair of primers in the region of the mutation (Table S1); the 500 bp PCR product was digested with Dde1 and the digestion products were separated on a 1% agarose gel (see Fig. 1). Mice were housed at the registered University of Barcelona animal facility, where they had *ad libitum* access to food (regular rodent chow) and water. They were maintained on a 12 h light/12 h dark (08:00–20:00 h/20:00−08:00 h) cycle. Both male and female mice were used in equal numbers in the experiments. All experimental procedures involving mice were validated by the University of Barcelona Ethics Committees on Animal Experimentation. These procedures were then approved by the Autonomous Government of Catalunya, in accordance with Spanish (RD 53/2013) and European legislation.

### Mouse phenotyping and functional assays

Mouse body weight was measured monthly on the same day. Forelimb maximal strength was assessed using a Grip Strength Meter (IITC Life Science, ALMEMO 2450 AHLBORN, World Precision Instruments, Hertfordshire, UK) with ten measurements per mouse, averaged and normalized to body weight (https://www.treat-nmd.org/wp-content/uploads/2023/07/MDX-DMD_M.2.2.001.pdf).

### Whole-body plethysmography

Respiratory function was assessed using whole-body plethysmography (WBP) with an EMMS instrument (EMMS, Waterlooville, UK) following standard procedures (https://www.treat-nmd.org/wp-content/uploads/2023/07/MDX-DMD_M.2.2.001.pdf) and the manufacturer's guidelines. In brief, mice were placed in calibrated chambers containing a pneumatograph that measured pressure differentials within the compartment caused by variations in airflow. The animals were allowed to acclimatize in the chambers for 45 min at a stable temperature and humidity (24ºC and 60%, respectively). Data were collected every 5 s using eDacq software (EMMS). Pause and enhanced pause (Penh) were defined and calculated by using the following equations: pause=(expiratory time−average relaxation time) ÷ average relaxation time and pause and enhanced pause=(peak expiratory pressure÷peak inspiratory pressure)×pause.

Final values for each parameter were derived from an average of 60 recordings, each one lasting 5 s, representing a total of 5 min.

### Tissue collection and processing

Mice (between two and five mice per sex and genotype) were euthanatized by CO_2_ inhalation followed by cervical dislocation. Dissection and morphological analysis of tissue and muscle samples were performed by veterinary pathologists trained to evaluate mouse tissues. Medial laparotomy and thoracotomy were performed, and liver, spleen, both kidneys, lung, heart and brain were dissected and weighed per mouse. The relative weight of each organ was calculated from body and brain weights. Hindlimb and forelimb muscles were dissected and weighed. Muscles were either frozen in isopentane pre-chilled on dry ice (for histology and immunofluorescence analysis) or snap-frozen in liquid nitrogen (for RNA and protein processing) and stored at −80°C.

### Histology

Hematoxilin and eosin (H&E) staining was conducted on 10-μm cryosections following standard procedures according to Treat-NMD (https://www.treat-nmd.org/wp-content/uploads/2023/07/cmd-MDC1A_M.1.2.004-68.pdf) For staining with Picro Sirius Red (Sigma-Aldrich, MA, USA), sections were incubated in xylene for 10 min and then passed quickly through 100%, 90% and 70% ethanol solutions. Then, sections were incubated in Picro Sirius Red solution containing 0.1% (w/v) of Direct Red 80 in picric acid for 60 min, washed twice with 2% acetic acid (v/v) and dehydrated serially in graded ethanol, cleared in xylene and mounted in DPX (Sigma-Aldrich). Histological preparations were imaged on a Nanozoomer S60 (Hamamatsu).

### RNA isolation and reverse transcription

Extraction and purification of total RNA from cell cultures were performed with the RNeasy^®^ Fibrous Tissue Mini Extraction Kit (Qiagen, Hilden, Germany). RNA concentration was measured as for DNA. Samples were stored at 80°C. For reverse transcription, equal amounts of RNA (300 ng) were used and added to an M-MLV reverse transcriptase reaction mix (Promega, Madison, WI, USA).

### Digital droplet PCR

Digital droplet PCR (ddPCR) was performed as previously described (López-Márquez et al., 2022) in a reaction volume of 20 μl using SuperMix for Probes (no dUTP) (Bio-Rad, Hercules, CA, USA), 450 nM of each primer pair, 250 nM of each probe (Table S1) and 0.025 or 2 ng of cDNA, depending on whether allele-specific or total expression was measured. PCR cycling conditions were optimized for each primer pair. Data were analyzed using Bio-Rad QuantaSoftTM software (v1.7.4), (Bio-Rad) with default settings for threshold determination to distinguish positive and negative droplets. The wild-type and mutant alleles were distinguished by a 2-dimensional view of the ddPCR analysis. Expression levels are reported as copies/μl and were normalized to *Gapdh*. At least three biological replicates were carried out for each experiment.

### Protein isolation, quantification and analysis

By using a Tissue Ruptor (Qiagen), muscle tissue (∼20−60 mg) was mechanically homogenized in lysis buffer containing 75 mM Tris-HCl (pH 6.8), 10% SDS, NAF (10 mM), Na_3_VO_4_ (1 mM) and a cocktail of protease inhibitors (Roche, #04693159001). Then we centrifuged the lysates (14000 ***g***, 4°C ) for 15 min. Protein concentration in the lysates was measured using the Pierce BCA Protein Assay (Thermo Scientific, Waltham, MA, USA; #23223 and #23224). Protein lysates (40 µg per lane) were separated under reducing conditions on a Bis-Tris polyacrylamide precast gel (4–12%) (Bio-Rad). Blotted membranes were blocked for 1 h in 5% milk or BSA in Tris-buffered saline [10 mM Tris, 150 mM NaCl (pH 8.0)] plus 0.1% Tween and incubated overnight at 4°C with rabbit polyclonal anti-collagen VI (Fitzgerald, #70R-CR009X) or rabbit polyclonal anti-desmin (Abcam, #ab8592) antibodies. Membranes were washed and incubated for 1 h at room temperature with peroxidase-conjugated secondary antibodies (1:10.000, Jackson ImmunoResearch Laboratories, USA). Bands were revealed with the ECL chemiluminescence detection system (Thermo Scientific) using the iBright detection equipment (Invitrogen) and the density of bands quantified using Fiji ImageJ software. The density of the bands corresponding to the collagen alpha-1 (VI) chain was normalized to the density of the desmin band for each sample, and data were reported as normalized values in arbitrary units (a.u.).

### Immunofluorescence

Cryosections of skeletal muscles (10 μm) were fixed in 4% paraformaldehyde for 10 min at room temperature and washed twice in PBS/glycine (0.1 M) solution, followed by two washes in PBS only, before blocking in 5% normal goat serum containing 0.5% Triton X-100 in PBS for 60 min. A mixture of rabbit polyclonal anti-collagen VI (Abcam, #ab6588; 1:1000) and rat-anti-perlecan (Merck, #MAB1948; 1:5000) primary antibodies in blocking solution was added to the sections, and kept overnight at 4°C. Sections were washed three times in PBS and incubated with secondary antibodies (Alexa Fluor-488-conjugated goat anti-rabbit IgG, 1:500 and Alexa Fluor-594-conjugated goat anti-rat IgG, 1:500) in blocking solution containing DAPI for 30 min at room temperature. Finally, sections were washed three times with PBS and mounted with Fluoromount-G mounting medium (Thermo Fisher Scientific, MA, USA).

### Automated muscle tissue mapping by using confocal microscopy

Confocal microscopy analysis was conducted using a Leica TCS SP8 STED 3X system, equipped with a white light laser, HyVolution mode and hybrid detectors (Leica Microsystems, Wetzlar, Germany). A high-precision motorized stage, controlled by LASX Navigator software, was employed to capture large-scale 3D mosaics of tissue sections at low magnification (HC PL APO CS2 10×/0.4 dry objective). The software automatically calculated the optimal stage positions based on the image dimensions in microns and the degree of overlap between adjacent images, ensuring complete coverage of the volume of interest. Individual image tiles were captured at 1024×1024 pixels with a *z*-step of 5 μm and 12 bits; between four and 20 stacks were collected for each extended image. Muscle tissues were sequentially excited at three wavelengths: 405 nm (DAPI), 488 nm (COL6) and 594 nm (Perlecan). Detection ranges were 420–465 nm for DAPI, 500–560 nm for COL6 and 610–755 nm for Perlecan. Ten sections were acquired every 5 μm across the tissue thickness, and maximum projections were generated by using LAS AF™ software (Leica Microsystems, Heidelberg, Germany).

### Image analysis

For the analysis of muscle fiber morphology, and nuclei, collagen VI and perlecan immunofluorescence intensity, images were extracted using the Leica Application Suite X (LAS X) and processed by using the Cellpose algorithm (Stringer et al., 2021), which uses a convolutional neural network to automatically deblur, denoise and segment fiber. Post-processing steps removed irregularly shaped fibers, those with non-smooth borders and isolated fibers to ensure reliable segmentation. The post-processed segmentations were saved as masks and divided into patches to enhance data availability. For size analysis of muscle fibers and internal nuclei quantitation, the original images and masks were processed to calculate the minimum Feret diameter and internal nuclear density per fiber for each fiber within the patches. For intensity analysis, the original images and masks were imported into QuPath software (Bankhead et al., 2017), and fluorescence intensity was measured at the fiber periphery (basal lamina) within a 2-µm area. Results were exported to Excel, with scripts available upon request.

### Quantification of fibrosis

To quantify the fibrotic tissue area of the muscle sections, an automatic image analysis pipeline was programmed with FIJI-ImageJ Software (Schindelin et al., 2012). Given the irregularity of some muscle sections, analysis was performed by selecting specific regions of interest (ROIs) comprising 500×500 pixels. This process was done manually by moving a rectangular window over the muscle section. When the window was placed on top of a desired ROI, the algorithm automatically segmented the fibrotic area of the muscle using the Li algorithm. Then, the area of the segmented region and its percentage over the whole ROI were computed. This process can be repeated by manually by moving the window to select all muscle ROIs that must be quantified, in order to map the whole muscle (Schindelin et al., 2012). Depending on size of the muscle section, between 41 and 176 ROIs were analyzed.

### Statistical analysis

To analyze body weight, piece-wise regression was used to study the age at which weight growth changes, after visually detecting this tendency with the corresponding boxplots. After determining the age range for these two time periods with clear different weight evolutions, they were analyzed separately. In both cases, linear regression models were used to study how age, sex and genotype explained weight for all data. This analysis was also performed separately for either sex. WBP data were analyzed with linear mixed models, in which age, sex and genotype were included as fixed effects, and random effects associated to subject ID were defined. Normality (QC plots) and homoscedasticity (residuals versus fitted values plots) in the residuals from these models were assessed using robust methods to create linear mixed models in cases where the residuals showed problems in the model fitting. *P*<0.05 or less was considered significant. Bonferroni−Holm correction for multiple testing was applied to the results from the WBP models. For morphometric analysis of muscle fibers (minimum Feret diameter and number of internal nuclei) and analysis of intensity of collagen VI and perlecan immunofluorescence, numerical variables and appropriate descriptive statistics were used. For each variable, different genotype groups were compared using Kruskal–Wallis test or pairwise Mann–Whitney *U*-test with Bonferroni−Holm adjustment as post-hoc analysis. The effect size (r) was used as the parameter for these comparisons. Significant results with r>0.1 were considered relevant and were discussed. Differences between groups regarding the distribution of minimum Feret diameter were studied by comparing 95% confidence intervals of the variance coefficient of each group.

One-way ANOVA test was used followed by Tukey's honestly significant difference (HSD) test as a post-hoc test to determine statistical significance for comparison weights of isolated muscles, grip strengths tests, digital PCRs, western blots and fibrosis values in percent. Statistical analyses and graphs were performed using R (v. 4.3), working with RStudio (v. 2022.2) or GraphPad PRISM Version 10.4.0.

## Supplementary Material

10.1242/dmm.052460_sup1 Supplementary information

Table S2. Summary of the statistical analysis of body weight and plethysmographic data.

Table S3. Statistical analysis of the plethysmographic data.
