# Supplementary material for: Col6a1 knock-in mice provide a promising pre-clinical model for collagen VI-related dystrophies
Source: Dis Model Mech. 2026 Jan 22;19(1):dmm052460. doi: 10.1242/dmm.052460 (PMC12869505; doi:10.1242/dmm.052460)
Supplement: Supplementary information [file dmm-19-052460-s1.pdf]

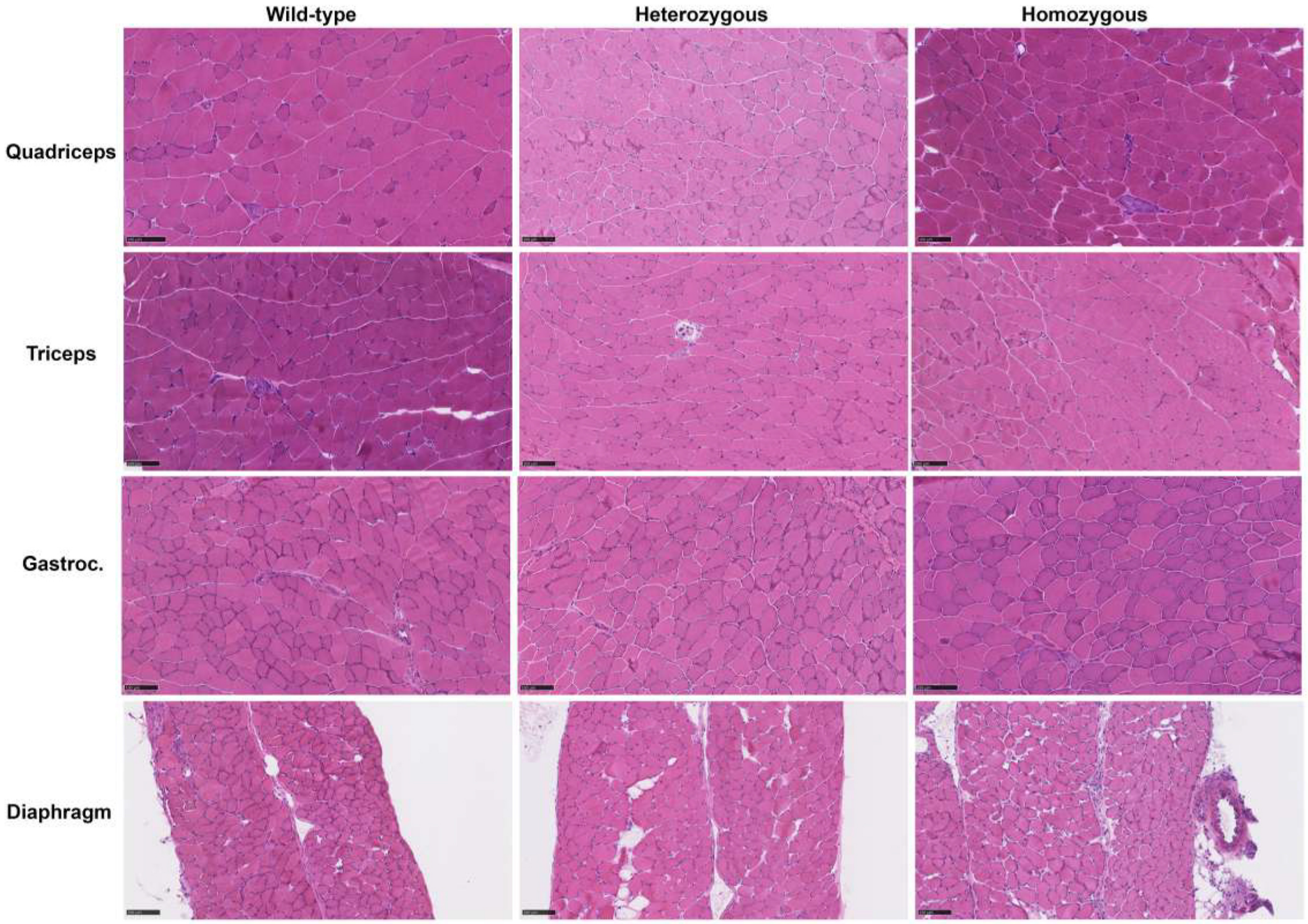

Fig. S1. Hematoxilin and eosin (H&E) staining of muscle sections as indicated, obtained from 12-months old mice.Scale bars = 100µm.

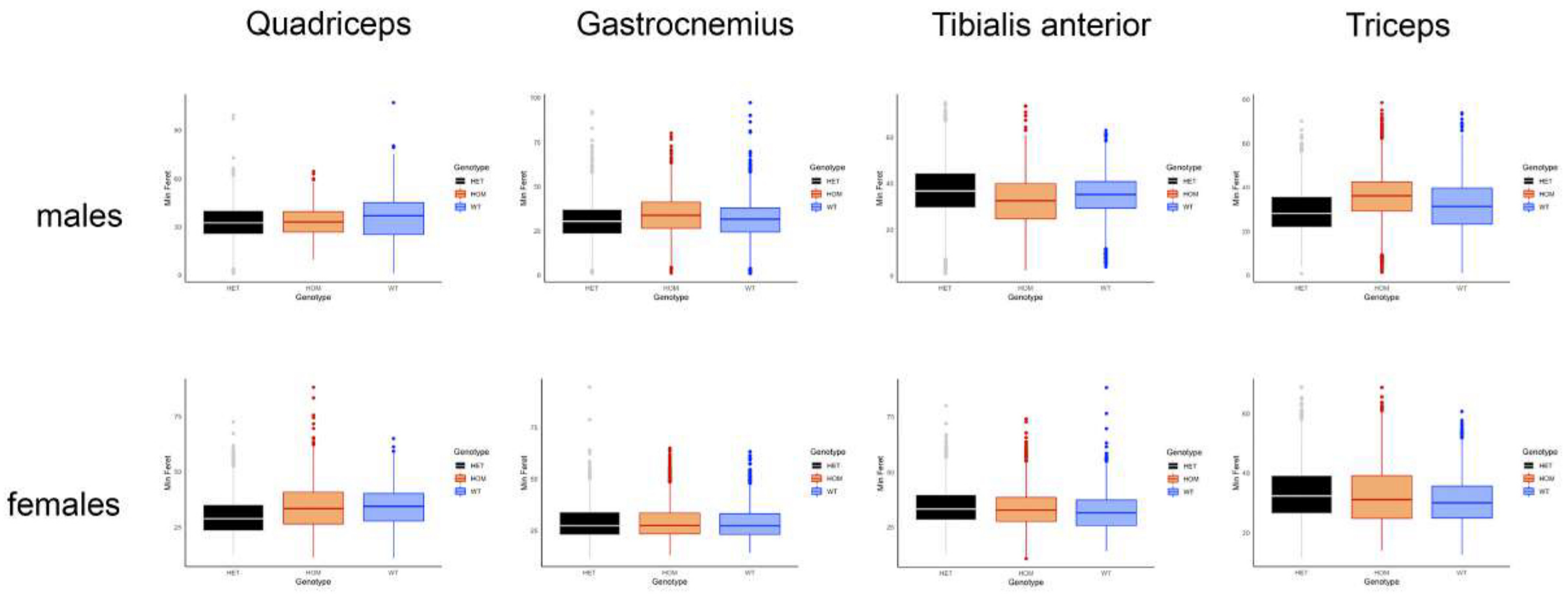

Fig. S2. Box plots representing the distribution (median and interquartile ranges) of the Minimum Feret diameter for different 6-months old male and female mice (analysed separately). The results of the statistical analysis (p-values and r-coefficients) for each comparison are provided in Table S4.

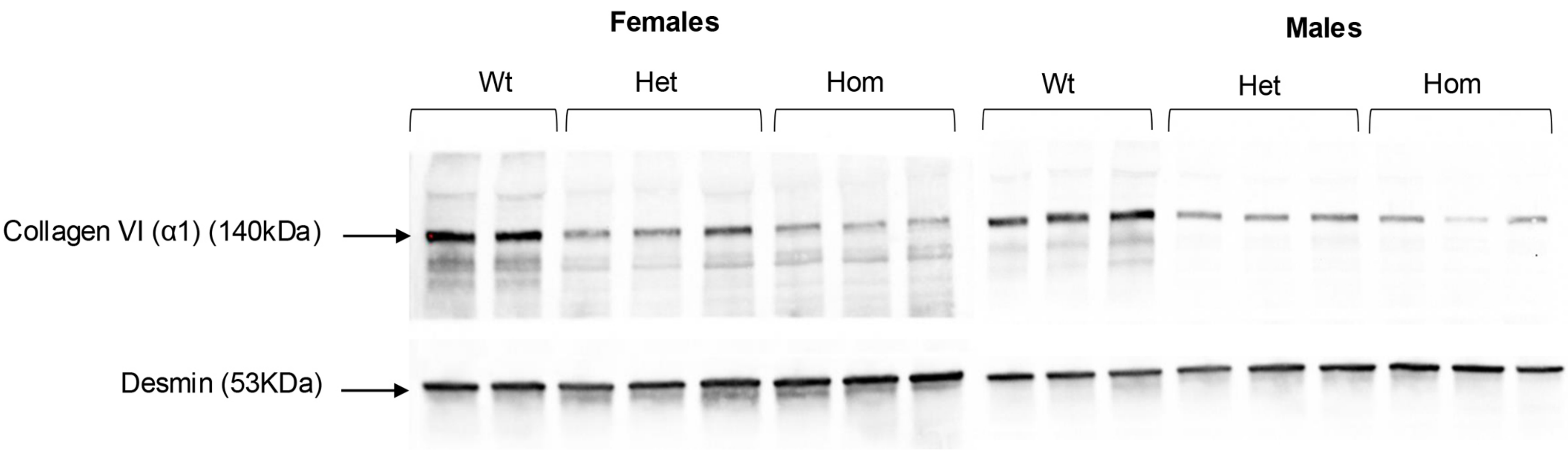

Fig S3. Representative images of a Western blot to detect collagen VI α1, (MW 140kDa), and desmin as the loading control (MW 53kDa), in diaphragm tissue extracts from female and male wild-type (wt) mice, heterozygous (het) and homozygous Col6a1 Ki G292R mice.

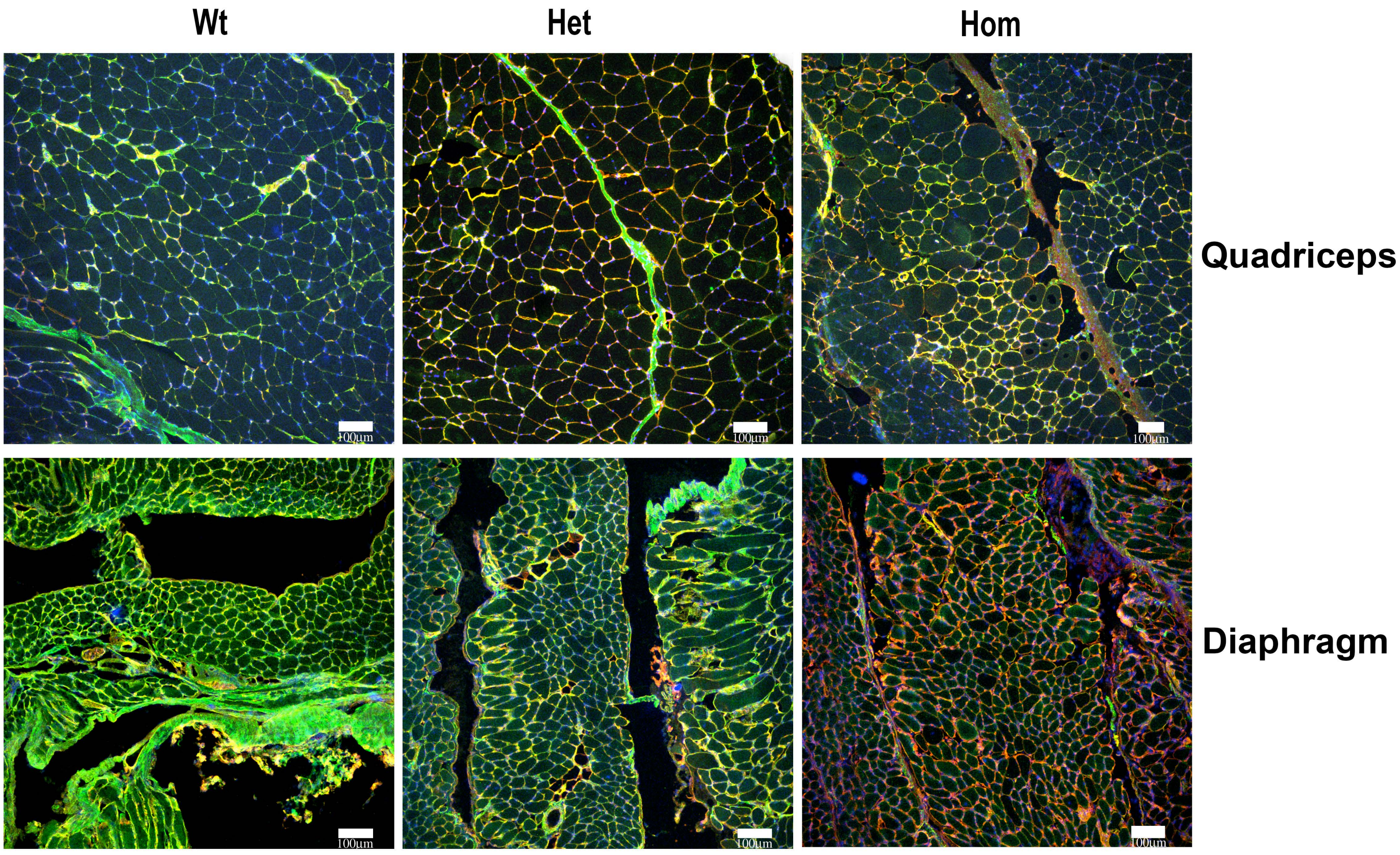

Fig. S4. Merged immunofluorescence images for collagen VI (green), perlecan (red) and the nuclei (blue) that are shown in Fig 7.

Disease Models & Mechanisms • Supplementary information

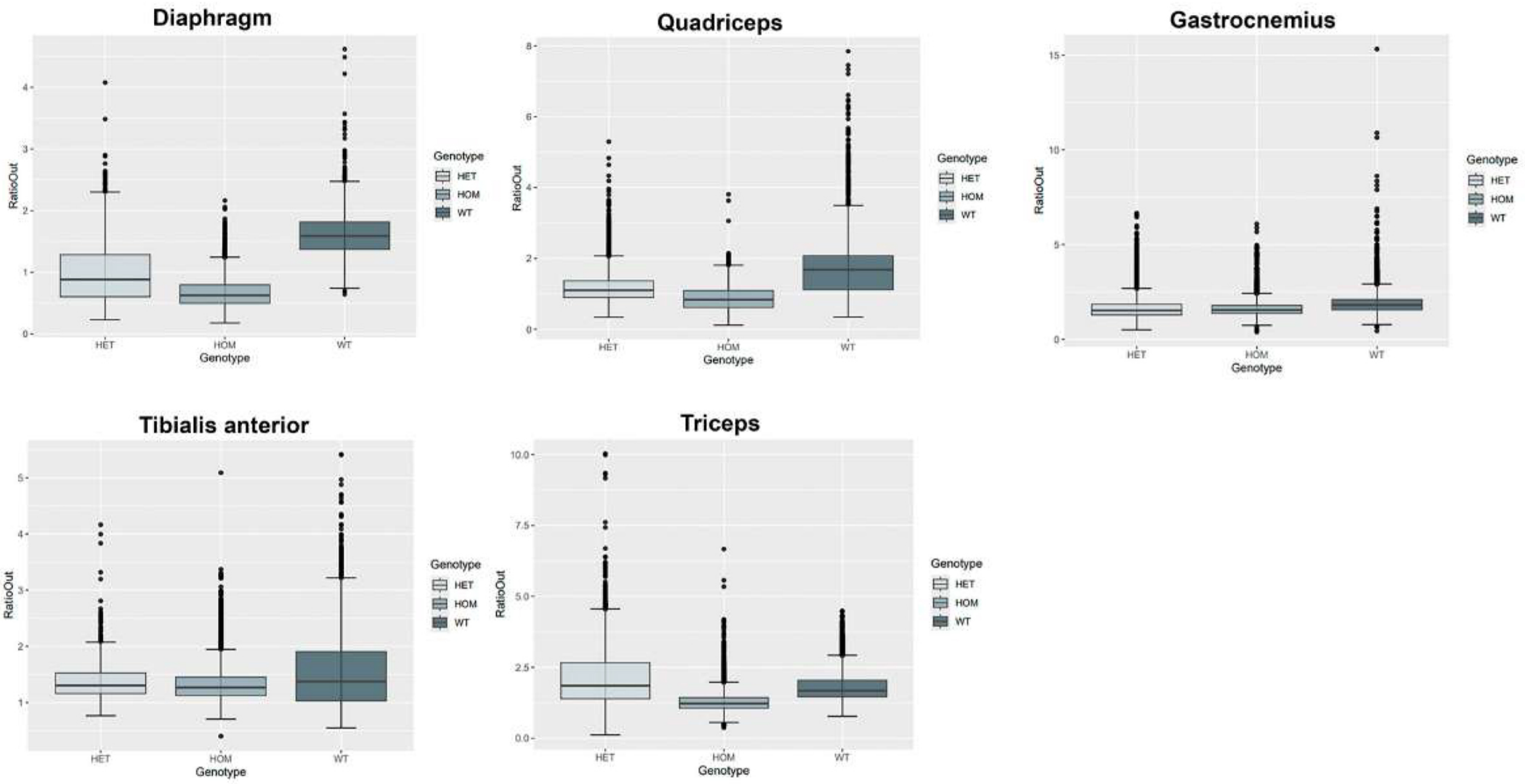

Fig. S5. Box plot of collagen VI/perlecan ratios (median and interquartile range) in arbitray units.

Table S1. Sequences and references for primers, gene expression assays and oligonucleotides. The sequence of the primers used to genotype mice by PCR as well as the sequence of the primers and probes (FAM or HEX labelled) used to amplify and detect the wild-type and mutant alleles by ddPCR is indicated. For *Col6a* transcripts, we used Gene Expression Assays by ThermoFsher Scientific (Inventoried Best Coverage) that contain the forward and reverse primers and the corresponding probes (FAM labelled). We also indicate the sequence of the single-stranded DNA template (ssODN) that was used to generate the col6a1 Ki mice.

**Gene/allele**	**Primer**	**Sequence (5´ to 3´)**	**Probe/allele**	**Sequence (5´ to 3´)**
Col6a1 genotyping (Salamanca)	Forward	CTCCCTTAAGGATTCTGTGTGG		
Col6a1 genotyping (Salamanca)	Reverse	GAGAAGGCAAGGAGGGTCTTC		
Col6a1 genotyping (Barcelona)	Forward	GGTGCATAACCTCTCCTCCA		
Col6a1 genotyping (Barcelona)	Reverse	AGAAGGCAAGGAGGGTCTTC		
col6a1 wild type/mutant	Forward	CGAGGAAAGCCAGGTCTTC	Wild type	ATCTTGGACCAGT (HEX)
col6a1 wild type/mutant	Reverse	TTCTCCCTTCATACCCTGGTA	Mutant	ATCTTAGACCAGTC (FAM)
**Gene**	**Gene Expression Assay (ref)**	**Sequence (5 ´to 3´)**		
Col6a1	Mm_00487160_m1	N/A		
Col6a2	Mm_00521578_m1	N/A		
Col6a3	Mm_00711678_m1	N/A		
Col6a5	Mm_01231908_m1	N/A		
Col6a6	Mm_00556810_m1	N/A		
Gapdh	Mm99999915_g1	N/A		
**Gene/allele**	**ssODN**	**Sequence (5´to 3´)**		
**col6a1**		CTGTAGCAAGGGGTAAGGTGGGCTGAGAAGGACCTTCCAGTGCTATCCAGGGATGTCT GACTATCTCTTGTTGTGTTCCAGGGACGACCTGGCGATCTTAGACCAGTCGGGTATCAG GGTATGAAGGTACGTGTCCTTAATGTCCAATGGCCTCTCTTGGGTGGGGTGGATCAGGC TTTGGAAGCAAAGCCCAAGCTGCC		

Table S2. Summary of the statistical analysis of body weight and plethysmographic data. Linear regression was used for body weight models and linear mixed models was used for plethysmographic data. Age, sex and genotype were included as independent variables in these models; the subject ID was used to define random effects in the mixed models. *P*-values of *P*<0.05 or less after Bonferroni-Holm correction for multiple testing was considered significant.

Available for download at
https://journals.biologists.com/dmm/article-lookup/doi/10.1242/dmm.052460#supplementary-data

Table S3. Statistical analysis of the plethysmographic data. EEP Average, end-expiratory pause; EIP Average, end-inspiratory pause; F Average, average respiratory rate; Pef Average, Peak Expirarory Flow; Penh Average, average pause and enhanced pause; Pif Average, average peak inspiratory flow; TE Average, expiratory time; TI Average, inspiratory time; tR Average, average relaxation time; TV, tidal volume; TV/Weight, tidal volume/weight.

Available for download at
https://journals.biologists.com/dmm/article-lookup/doi/10.1242/dmm.052460#supplementary-data

Table S4. Analysis of the Minimum Feret´s diameter for 6 months old male (A) and female mice (B). We used Kruskal-Wallis test to compare the values from these 3 groups, using Mann-Whitney's U-test with Bonferroni-Holm correction for multiple testing as post-hoc analysis. The p value is indicated for the comparison between Wild-type (wt) and heterozygous (het) in the het column and for the comparison between wt and hom (hom) in the hom column and in brackets the value for the comparison het vs hom. We also calculate the r parameter as an effect size measure for these tests, as we have a large sample size,and it is relatively easy to obtain significant results with really small differences between groups. * An r (effect sizes) equal or larger than 0.1is considered relevant. The r parameter is indicated for the comparison between wt and het in the het column and for the comparison between wt and hom in the hom column and in brackets the value for the comparison het vs hom.

A. Male

	GSN			QUA			TA			TRI		
	Wt	Het	Hom	Wt	Het	Hom	Wt	Het	Hom	Wt	Het	Hom
**Mean**	30.8	30.1	33.5	34.4	32.1	33.2	34.9	36.3	31.9	31.4	28.8	35.4
**SD**	10.4	10.2	11.1	14.5	11.6	8.35	8.69	12.1	12.3	11.3	10.3	10.6
**p-value**		<0.0001	<0.0001 (<0.0001)		<0.0001	<0.0001 (0.0001)		<0.0001	<0.0001 (0.0001)		<0.0001	<0.0001 (0.0001)
**r**		0.041	0.112 (0.135)		0.119	0.105 (0.031)		0.076	0.107 (0.113)		0.118	0.188 (0.32)

B. Female

	GSN			QUA			TA			TRI		
	Wt	Het	Hom	Wt	Het	Hom	Wt	Het	Hom	Wt	Het	Hom
**Mean**	28.8	28.8	29.0	34.0	29.9	34.1	32.1	34.2	33.1	30.4	32.9	32.5
**SD**	7.79	7.77	7.77	8.23	9.73	8.38	8.84	8.96	8.04	7.53	9.89	8.71
**p-value**		ns	ns(ns)		<0.0001	0.067 (0.0001)		<0.0001	<0.0001 (0.0001)		<0.0001	<0.0001 (0.0001)
**r**					0.258	(0.217)		0.115	0.065 (0.038)		0.136	0.076 (0.041)

Table S5. Coefficient of variation (CV) for the Minumum Feret´s diameter (mean and 95% confidence interval) for the different muscles analysed from 6 months old mice. Only muscles where there was no overlap in the CI between genotypes were considered relevant.

Males

Gastrocnemius

WT

```
##      CV         low.ci       upr.ci
## 0.3379708   0.3328758    0.3432415
```

HET

```
##      CV         low.ci       upr.ci
## 0.3378576   0.3336940    0.3421378
```

HOM

```
##      CV         low.ci       upr.ci
## 0.3311452   0.3238741    0.3387879
```

Quadriceps

WT

```
##      CV         low.ci       upr.ci
## 0.3379708   0.3328758    0.3432415
```

HET

```
##      CV         low.ci       upr.ci
## 0.3613936   0.3541501    0.3689723
```

HOM

```
##        CV       low.ci       upr.ci
## 0.2511929   0.2456909    0.2569775
```

Tibialis anterior

WT

```
##      CV         low.ci       upr.ci
## 0.2489178   0.2428269    0.2553605
```

HET

```
##      CV         low.ci       upr.ci
## 0.3338267   0.3280307    0.3398543
```

HOM

```
##      CV          low.ci      upr.ci
## 0.3836996   0.3645574    0.4052032
```

Triceps

WT

CV low.ci upr.ci

0.3611101 0.3549884 0.3674697

HET

CV low.ci upr.ci

0.3572489 0.3499408 0.3649026

Females

Gastrocnemius

WT

CV low.ci upr.ci

0.2700564 0.2629435 0.2776126

HET

CV low.ci upr.ci

0.2700904 0.2656587 0.2746901

HOM

CV low.ci upr.ci

0.2682083 0.2632063 0.2734271

Quadriceps

WT

CV low.ci upr.ci

0.2418628 0.2360415 0.2480148

HET

CV low.ci upr.ci

0.2802239 0.2735422 0.2872801

HOM

CV low.ci upr.ci

0.2856936 0.2798578 0.2918072

Tibialis anterior

WT

##	CV	low.ci	upr.ci
##	0.2752358	0.2653322	0.2860017

HET

##	CV	low.ci	upr.ci
##	0.2620751	0.2546228	0.2700317

HOM

##	CV	low.ci	upr.ci
##	0.2426263	0.2381267	0.2473204

Triceps

WT

##	CV	low.ci	upr.ci
##	0.2474159	0.2422706	0.2528117

HET

##	CV	low.ci	upr.ci
##	0.2648826	0.2598880	0.2700963

HOM

##	CV	low.ci	upr.ci
##	0.2648826	0.2598880	0.2700963

Disease Models & Mechanisms • Supplementary information

Table S6. Analysis of the collagenVI/perlecanfluorescence intensity ratio between genotypes in 12 months old mice. Mean intensity in a.u. (standard deviation) is indicated for each genotype. We used Kruskal-Wallis test to compare the values from these 3 groups, using Mann-Whitney's U-test with Bonferroni-Holm correction for multiple testing as post-hoc analysis. The p value is indicated for the comparison between Wild-type (wt) and heterozygous (het) in the het column and for the comparison betweenwild-type andhomozygous (hom) in thehomcolumn and in brackets the value for the comparison het vshom. We also calculate the r parameter as an effect sizemeasure for these tests, as we have a large sample size,and it is relatively easy to obtain significant results with really small differences between groups. * An r (effect sizes) equal or larger than 0.1is considered relevant. The r parameter is indicated for the comparison between wt and het in the het column and for the comparison betweenwt and hom in the hom column and in brackets the valuefor the comparison het vs hom. Only r values = or > than 0.1 are indicated. Fold change between wt and the other genotypes is also indicated (wt/het or hom).

	DIA			Quads			GSN			TA			Triceps		
	Wt	Het	Hom	Wt	Het	Hom	Wt	Het	Hom	Wt	Het	Hom	Wt	Het	Hom
Mean intensity (a.u) (SD)	1.61 (0.35)	0.97 (0.42)	0.67 (0.23)	1.64 (0.67)	1.16 (0.37)	0.87 (0.31)	1,87 (0,52)	1,62 (0,49)	1,6 (0,38)	1,52 (0.6)	1.37 (0.32)	1.33 (0.3)	1.80 (0.48)	2(0.9)	1.28 (0.34)
p-value		< 0,001	< 0,001 (<0,001)		< 0,001	< 0,001 (< 0,001)		< 0,001	< 0,001 (<0,001)		< 0,001	< 0,001 (<0,001)		< 0,001	< 0,001 (<0,001)
r-value *		0.56	0.76 (0.37)		0.39	0.6 (0.4)		0,3	0,32					0.1	0.58 (0.47)
Fold change		1,66	2,4		1,4	1,9		1,15	1,17					-1.1	1.4

Disease Models & Mechanisms • Supplementary information
